# Differential effects of PDE5 inhibitors on cardiac dysfunction in the MDX ouse model of Duchenne muscular dystrophy

**DOI:** 10.1186/2050-6511-14-S1-O38

**Published:** 2013-08-29

**Authors:** Sergei D Rybalkin, Masami Shimizu, Irina G Rybalkina, Enrico Patrucco, Kenneth Bible, Elina Minami, Jennifer O'Brien, Lawrence P Wennogle, Franz Hofmann, Joseph A Beavo, Stanley C Froehner

**Affiliations:** 1 Departments of Pharmacology, University of Washington, Seattle, WA 98195, USA; 2 Institut für Pharmakologie und Toxikologie, TU, München, Germany; 3 Department of Physiology and Biophysics, University of Washington, Seattle, WA 98195, USA; 4 Departments of Cardiology, University of Washington, Seattle, WA 98195, USA; 5 Intra-Cellular Therapies, Inc, New York , NY 10032, USA

## Background

Duchenne muscular dystrophy (DMD) is the most common inherited form of muscular dystrophy, which results in skeletal muscle weakness by age 6. In its later stages DMD leads to dilated cardiomyopathy and heart failure with high level of mortality. At present there are no effective treatments for most of the cardiac pathology in DMD patients.

## Results

Recently we showed that sildenafil could reverse much of the cardiac dysfunction in the mdx mouse model of DMD. Treatments with sildenafil (in the drinking water) resulted in significant improvements in cardiac function, analyzed by the myocardial performance index (MPI) and Ea/Aa ratios [1]. Similar improvements in cardiac functions were also observed when sildenafil was administered daily for one week by oral gavage (60 mg/kg). However, daily administration of tadalafil by oral gavage (4 and 20 mg/kg) did not produce any improvement in the myocardial performance index.

In order to provide a reliable indicator for efficacy of dosing of different PDE5 inhibitors we examined the levels of PDE5 phosphorylation in tissues known to express PDE5, e.g. lung and blood vessels, as a biological indicator of cGMP induced PKG activation in vivo. We found that both tadalafil and sildenafil caused time-dependent equivalent phosphorylation of PDE5 in these tissues. Similar PDE5 phosphorylation patterns were observed in cardiac tissue extracts. Since no PDE5 expression has been detected in adult mouse cardiac myocytes, we consider phospho-PDE5 in the cardiac samples to be from cardiac blood vessels, myofibroblasts and even platelets from residual blood.

Differential effects of PDE5 inhibitors on cardiac dysfunctions in MDX mice could point to PDE1C, which can be partially inhibited by sildenafil, but not by tadalafil. PDE1C is the calcium/calmodulin cGMP/cAMP PDE, most highly expressed in mouse cardiac myocytes. However, the functions of PDE1C in myocytes have not been determined.

We used specific PDE1 inhibitors (Intra-Cellular Therapies, NY) and found that these inhibitors could substantially stimulate cGMP-induced phosphorylation of phospholamban by C-type natriuretic peptide (CNP). Moreover, in cardiac myocytes, isolated from PDE1C KO mice, CNP-induced phosphorylation of phospholamban was substantially higher, and no additional increase of its phosphorylation was detected with PDE1 specific inhibitors.

However, we did not detect any induction of phosholamban phosphorylation when sildenafil was applied to mouse cardiac myocytes even at high concentrations; and tadalafil did not have any effects as well. Although the pattern of PDE5 phosphorylation after sildenafil and tadalafil application by oral gavage corresponded to the differences in the pharmacokinetics of these drugs, they produced differential changes in ERK, VASP and GSKb phosphorylation in lung and heart, often in different directions.

## Conclusion

These data suggest that the sildenafil and tadalafil differential effects could be the result of indirect effects of these drugs on other cell types, subsequently affecting cardiac functions.

However, PDE1 specific inhibitors appear to be new potential agents for direct regulation of phospholamban phosphorylation and calcium homeostasis in the heart.
